# An egg a day could significantly reduce CVD risk

**Published:** 2018

People who consume an egg a day could significantly reduce their risk of cardiovascular disease (CVD) compared with eating no eggs, suggests a study carried out in China. CVD is the leading cause of death and disability worldwide, including China, mostly due to ischaemic heart disease and stroke (including both haemorrhagic and ischaemic stroke).

Unlike ischaemic heart disease, which is the leading cause of premature death in most Western countries, stroke is the most responsible cause in China, followed by heart disease. Although ischaemic stroke accounted for the majority of strokes, the proportion of haemorrhagic stroke in China is still higher than that in high-income countries.

Eggs are a prominent source of dietary cholesterol, but they also contain high-quality protein, many vitamins and bioactive components such as phospholipids and carotenoids.

Previous studies looking at associations between eating eggs and impact on health have been inconsistent, and most of them found insignificant associations between egg consumption and coronary heart disease or stroke. Therefore, a team of researchers from China and the UK led by Prof Liming Li and Dr Canqing Yu from the School of Public Health, Peking University Health Science Centre, set out to examine the associations between egg consumption and cardiovascular disease, ischaemic heart disease, major coronary events, haemorrhagic stroke and ischaemic stroke.

They used data from the China Kadoorie Biobank (CKB) study, an ongoing prospective study of around half a million (512 891) adults aged 30 to 79 years from 10 different geographical areas in China. The participants were recruited between 2004 and 2008 and were asked about the frequency of their egg consumption. They were followed up to determine their morbidity and mortality.

For the new study, the researchers focused on 416 213 participants who were free of prior cancer, CVD and diabetes. From that group at a median follow up of 8.9 years, a total of 83 977 cases of CVD and 9 985 CVD deaths were documented, as well as 5 103 major coronary events. At the start of the study period, 13.1% of participants reported daily consumption of eggs (usual amount 0.76 eggs/day) and 9.1% reported never or very rare consumption of eggs (usual amount 0.29 eggs/day).

Analysis of the results showed that compared with people not consuming eggs, daily egg consumption was associated with a lower risk of CVD overall. In particular, daily egg consumers (up to one egg/day) had a 26% lower risk of haemorrhagic stroke, a 28% lower risk of haemorrhagic stroke death and an 18% lower risk of CVD death.

In addition, there was a 12% reduction in risk of ischaemic heart disease observed for people consuming eggs daily (estimated amount 5.32 eggs/week), when compared with the ‘never/rarely’ consumption category (2.03 eggs/week).

This was an observational study, so no firm conclusions can be drawn about cause and effect, but the authors said their study had a large sample size and took into account established and potential risk factors for CVD.

The authors concluded: ‘The present study finds that there is an association between moderate level of egg consumption (up to 1 egg/day) and a lower cardiac event rate. Our findings contribute scientific evidence to the dietary guidelines with regard to egg consumption for the healthy Chinese adult.’
